# Central Nervous System Injury in Patients With Severe Acute Respiratory Syndrome Coronavirus 2: MRI Findings

**DOI:** 10.7759/cureus.18052

**Published:** 2021-09-17

**Authors:** Edith Fabiola Mendez Elizondo, José Arturo Valdez Ramírez, Gustavo Barraza Aguirre, Paulette Mariette Dautt Medina, Jorge Berlanga Estens

**Affiliations:** 1 Radiology, American British Cowdray Medical Center, Mexico City, MEX; 2 Radiology, CT Scanner Lomas Altas, Mexico City, MEX

**Keywords:** central nervous system injury, magnetic resonance, sars-cov-2, neurological deterioration, infection

## Abstract

Due to the presence of a new and rapidly spreading coronavirus, severe acute respiratory syndrome coronavirus 2 (SARS-CoV-2), the World Health Organization declared the coronavirus disease 2019 (COVID-19) outbreak a pandemic on March 11, 2020. This new disease has a multisystemic effect that predominantly targets the respiratory system; however, neurologic symptoms have been documented in approximately 36% of patients with confirmed COVID-19. During the period of March 2020 to March 2021, 481 brain MRI studies were performed by medical request. Of these, 9.7% (n = 47) were hospitalized with a diagnosis of COVID-19 pneumonia confirmed by SARS-CoV-2 reverse transcriptase-polymerase chain reaction (RT-PCR) test with the following findings: microbleeds, osmotic demyelination, arterial thrombosis, ischemic infarcts, venous thrombosis, metabolic cerebellar syndrome, posterior reversible leukoencephalopathy, abnormal signal intensity in the frontal lobes and olfactory bulbs, microangiopathy, gliosis, and findings consistent with hypoxic-ischemic encephalopathy. In patients with histories of malignant central nervous system (CNS) tumors, the most frequent histological lineage being high-grade glioma, 100% progression was identified with respect to previous imaging studies, without other significant findings. In two patients, a brain MRI was performed due to altered alertness, identifying only involutive changes in the brain parenchyma; MRI was repeated 72 hours later, after a lack of improvement in higher functions, without identifying imaging findings. To date, limited studies have documented CNS abnormalities related to COVID-19 using MRI. Therefore, the purpose of this study is to present abnormal imaging findings in patients with SARS-CoV-2 infection and their clinical correlations.

## Introduction

The severe acute respiratory syndrome coronavirus 2 (SARS-CoV-2) emerged in China in the last two months of 2019 and caused a severe pandemic (coronavirus disease 2019 {COVID-19}), characterized by severe acute respiratory syndrome. COVID-19 has multisystemic involvement due to the virus binding to the cellular receptor for angiotensin-converting enzyme 2 (ACE2), which is found in multiple cells of the human body, including neuronal cells. The virus accesses neuronal cells by a retrograde neuronal pathway or hematogenous dissemination, which explains the neurological symptoms that have been reported in up to 36% of patients who have returned positive SARS-CoV-2 reverse transcriptase-polymerase chain reaction (RT-PCR) tests. The symptoms include disturbed consciousness, paresthesia, anosmia, and headache [1].

## Materials and methods

From March 2020 to March 2021, 481 brain MRI studies were performed on a Siemens MAGNETOM Skyra 3 Tesla resonator (SIEMENS S/N 145452, Software NUMARIS/4, Version syngo MR E11, Mexico City) with at least the following sequences: axial and coronal T2-weighted image (T2WI), axial T1-weighted image (T1WI), sagittal and coronal T1-fluid-attenuated inversion recovery (FLAIR), axial T2-FLAIR, axial diffusion-weighted imaging (DWI) and apparent diffusion coefficient (ADC) map, axial gradient echo sequences (GRE), and three-dimensional time of light (3D TOF). Other sequences such as susceptibility-weighted images (SWI) and post-contrast T1-weighted sequences were acquired at the request of the radiologist.

A clinical-radiological correlation was performed, and SARS-CoV-2-positive patients (confirmed by RT-PCR test) were selected. Image analysis was performed by three radiology specialists, and case follow-up was performed by an imaging resident. In addition, published scientific literature was reviewed.

## Results

Of the total number of patients who underwent an MRI study by medical request, 9.7% (n = 47) were hospitalized with a diagnosis of COVID-19 pneumonia confirmed by a SARS-CoV-2 RT-PCR positive (+) test result. Microbleeds were the only finding in 5.4% (n = 26) of the patients, and of these, 23.07% (n = 6) had a SARS-CoV-2 RT-PCR (+) test result. Microbleeds were also identified in a patient with osmotic demyelination and COVID-19 pneumonia. Three patients (0.62%) presented with arterial thrombosis and one with a SARS-CoV-2 RT-PCR (+) test result and extensive ischemic infarction development. Ischemic infarcts were identified in further three patients, resulting in a total of four patients with SARS-CoV-2 RT-PCR (+) test results. Four patients (0.83%) presented with venous thrombosis, and one of them had COVID-19 infection. Metabolic cerebellar syndrome associated with microangiopathy was diagnosed in only one patient who was SARS-CoV-2 RT-PCR (+). Abnormal signal intensity in the olfactory bulbs and findings consistent with hypoxic-ischemic encephalopathy were observed in two patients (one in each) with COVID-19. Also, we found two cases of posterior reversible encephalopathy syndrome (0.41%), one with a COVID-19 infection. Eight patients (1.6%) had histories of malignant CNS tumors, the most frequent histological lineage being high-grade glioma (n = 3, 37.5%) and 100% progression was identified with respect to a previous imaging study without other significant findings. In two patients, a brain MRI was performed due to altered alertness, identifying only involutive changes in the brain parenchyma; MRI was repeated 72 hours later, after a lack of improvement in higher functions, without identifying imaging findings. In the remaining patients with COVID-19 pneumonia confirmed by SARS-CoV-2 RT-PCR (+) test results and without any other coexisting disease that explained the neurological impairment, changes due to microangiopathy were identified in 16 patients (3.3%), and many areas of gliosis were diagnosed only in two patients.

## Discussion

Neurological symptoms have been reported in up to 36% of patients who have returned positive SARS-CoV-2 reverse transcriptase-polymerase chain reaction (RT-PCR) tests and these symptoms most likely have a morphological representation. Magnetic resonance imaging of the brain is a painless and safe test that uses a magnetic field and radio waves to produce detailed images of the brain can provide clear images of parts of the brain that can’t be seen as well with X-rays, tomography, or ultrasound studies in which the diagnosis is often missed. Brain abnormalities have been detected in patients infected with the new coronavirus causing the current pandemic, these findings are described below.

Frontal lobe findings

**Figure 1:**
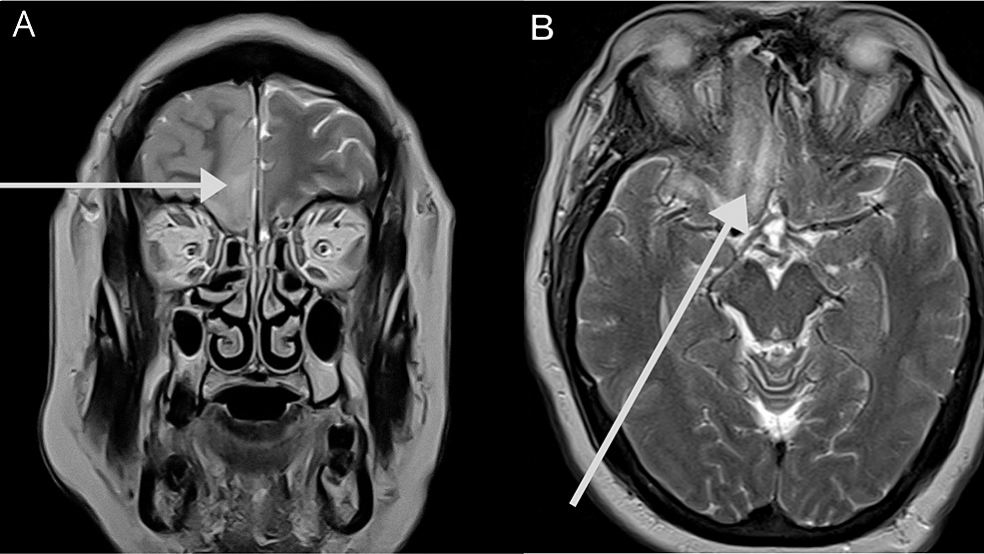
Olfactory neuropathy in a 47-year-old male patient with a stiff neck, headache, disorientation, anosmia, SARS-CoV-2 RT-PCR (+) test result, and pneumonia. (A) Coronal T2WI shows unilateral frontal cortical thickening and decreased subarachnoid space. (B) Axial T2WI with high signal intensity in the left olfactory bulb and ipsilateral gyrus rectus. SARS-CoV-2 RT-PCR (+): positive severe acute respiratory syndrome coronavirus 2 reverse transcriptase-polymerase chain reaction

Abnormal signal intensity (cortical hyperintensity in fluid sensitive sequences) in the gyrus rectus and olfactory bulbs is the most common finding (Figures 1A, 1B). The spin-echo images show asymmetry in olfactory bulb thickness, and T2WI with/without fat suppression revealed linear hyperintensities in one or both olfactory nerves, indicative of olfactory neuropathy [2].

Brain microbleeds

**Figure 2:**
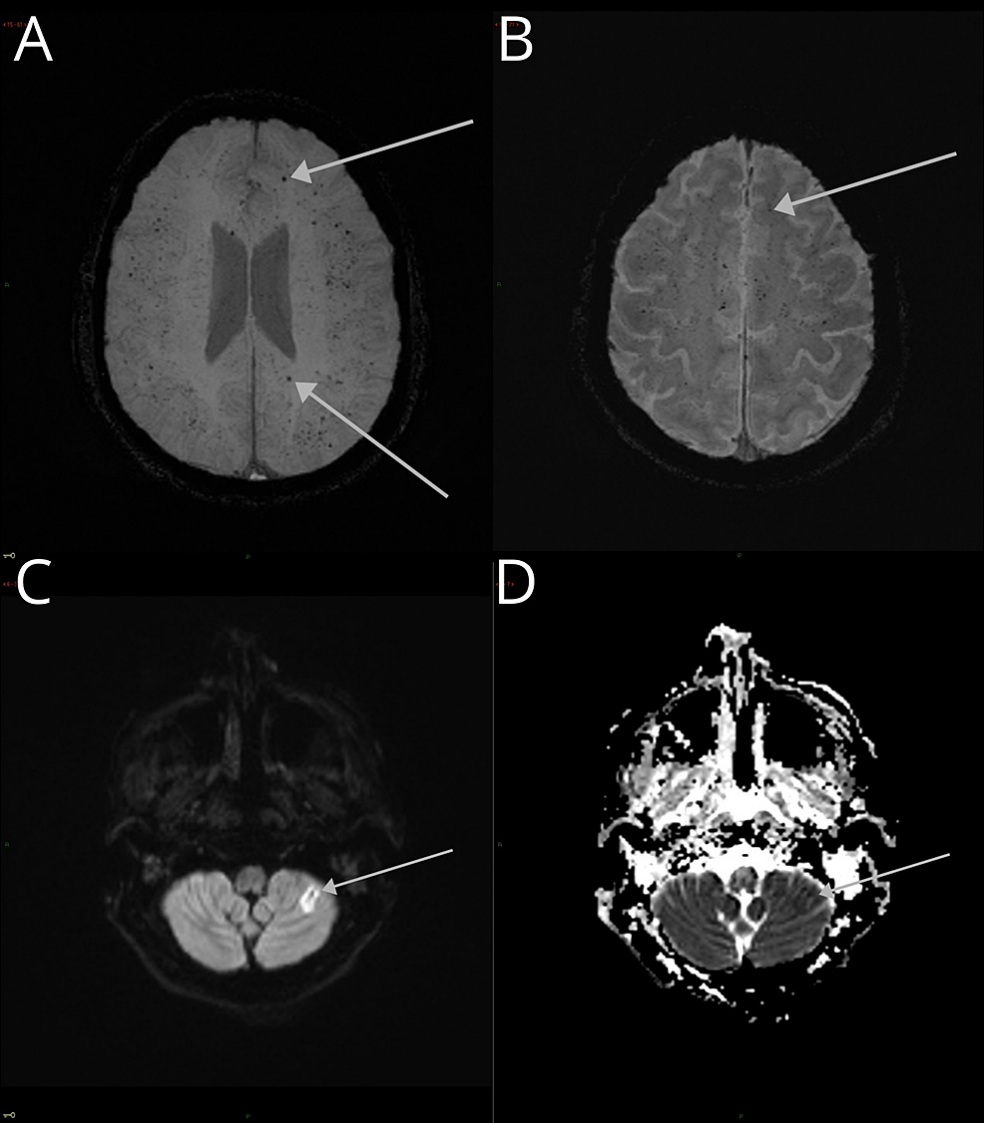
Brain microbleeds and ischemic cerebrovascular event in a 61-year-old male with a history of hypertension, diabetes, and dyslipidemia, developed severe COVID-19 pneumonia and neurological alterations (areflexia, absent conjugated gaze fixation, indifferent fixation, and an indifferent Babinski sign). (A and B) GRE and SWI, with an increased number of juxtacortical BMB. (C and D) DWI in the same patient shows a high-intensity signal on the posterior cerebellar lobe with a low signal on the apparent diffusion coefficient sequence illustrating an ischemic cerebrovascular event, demonstrating the overlap of pathologies. COVID-19: coronavirus disease 2019; BMB: brain microbleeds; GRE: gradient-echo sequences; SWI: susceptibility-weighted images; DWI: diffusion-weighted imaging

**Figure 3:**
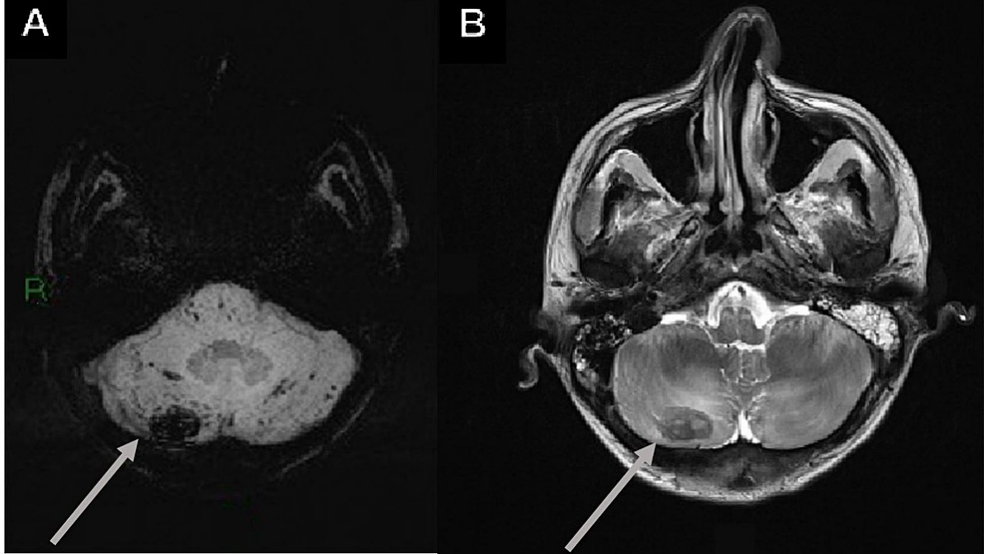
Cerebellar microbleeds and cerebellar hemorrhage in a 30-year-old patient. (A) SWI axial image with brainstem and cerebellar hemisphere microbleeds. (B) T2WI with right cerebellar hemorrhage and perilesional edema. SWI: susceptibility-weighted images; T2WI: T2-weighted image

**Figure 4:**
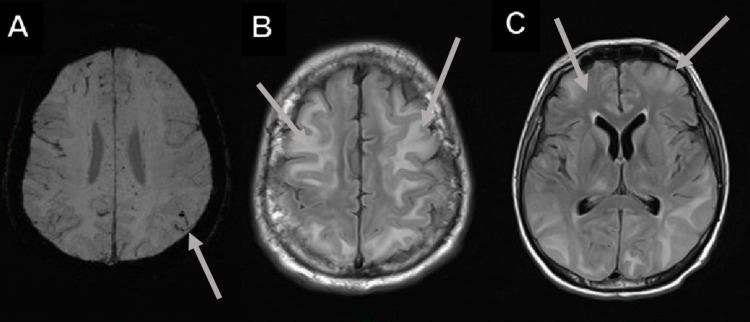
White matter BMB and images with suspected leukoencephalopathy. (A) SWI image with multiple juxtacortical and white matter BMB. (B and C) Subcortical bilateral asymmetrical hyperintensities in the T2-FLAIR images; suspected leukoencephalopathy. BMB: brain microbleeds; T2-FLAIR: T2-weighted fluid-attenuated inversion recovery

Brain microbleeds (BMB) are the result of small blood extravasations due to rupture of the wall of small capillaries, arterioles, or arteries, either due to lipohyalinosis, microaneurysm, or cerebral amyloid angiopathy (CAA). Lipohyalinosis resulted in BMB localized to the basal ganglia, thalamus, brainstem, and cerebellum, whereas CAA resulted in BMB localized to the cerebral cortex and/or juxtacortical (Figures 2A-2D), cerebellum (Figures 3A, 3B), and deep and periventricular white matter (Figures 4A-4C) [3].

A 61-year-old male with a history of hypertension, diabetes, and dyslipidemia, developed severe COVID-19 pneumonia and was intubated for a month. After sedation was tapered, the patient presented with spontaneous ocular opening with areflexia, absent conjugated gaze fixation, indifferent fixation, and an indifferent Babinski sign (Figure 2).

BMB can affect cognitive capacity, possibly through cortico-subcortical and intracortical disconnection, and are associated with increased mortality. BMB increase the risk of cerebral hemorrhage, especially in patients with multiple lobar BMB, so anticoagulant treatment may be contraindicated in these patients [3].

**Figure 5:**
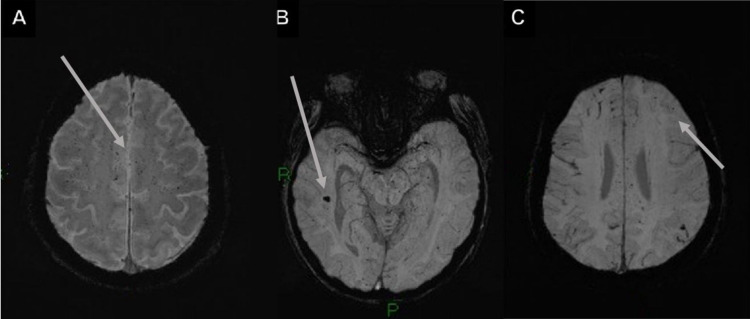
Findings in the SWI sequence of microbleeds. (A-C) Axial SWI images show innumerable microhemorrhages with diffuse and bilateral distribution, predominantly in the subcortical white matter and juxtacortical regions. (B) Microhemorrhages in the hippocampus and midbrain are also shown.

Microbleeds were defined by meeting the following criteria: small, well-defined, round, or ovoid in shape homogeneous hypointense lesions (black) on T2-GRE or SWI (Figures 5A-5C); “blooming” effect on T2-GRE and SWI images compared to T1- or T2-weighted sequences; absence of signal hyperintensity on T1WI or T2WI; and the lesion being surrounded by brain parenchyma (at least half of the lesion). The clinical history helped to differentiate BMB from other "microhemorrhage mimics" (hypointense lesions or artifacts) and excluded traumatic diffuse axonal injury in the absence of recent trauma [4].

Venous thrombosis

Cerebral venous thrombosis (CVT) is responsible for 1-2% of all strokes in adults and affects all age groups [5]. The presentation can be highly variable and range from essentially asymptomatic to coma and death, and the most common symptom is headache (89-91%) [6]. Imaging findings are secondary to intracranial hemorrhage or edema (cytotoxic or vasogenic) [7].

**Figure 6:**
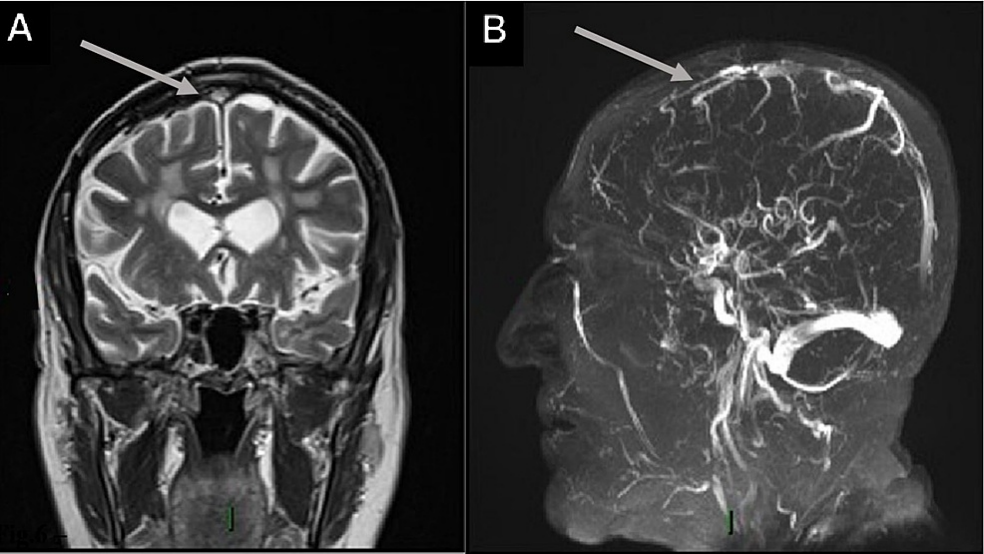
Superior sagittal sinus thrombosis in a 75-year-old male who was RT-PCR (+) for SARS-CoV-2 and had respiratory symptoms, a history of hypertension, and a prior meningioma resection with symmetrical leg weakness and loss of consciousness. (A) Coronal T2WI shows a high signal intensity image located on the superior sagittal sinus. (B) 3D TOF image with loss of flow signal in the rostral segment of the superior sagittal sinus. SARS-CoV-2 RT-PCR (+): positive severe acute respiratory syndrome coronavirus 2 reverse transcriptase-polymerase chain reaction; T2WI: T2-weighted image; 3D TOF: three-dimensional time of light

Unenhanced computed tomography (CT) is usually the first investigation performed [7]. The thrombus can appear as a hyperdense vein or sinus for the first seven to 14 days. This is an accurate sign when present, but this intravascular hyperattenuation has low sensitivity since it is only found in one-quarter of cases [8]. In such cases, the patient must be evaluated with MRI or contrast-enhanced tomography. There was greater involvement of the superior sagittal sinus (60%), left transverse sinus (45%), right transverse sinus (40%), and, to a lesser extent, the straight sinus, cavernous sinus, and deep cortical veins (Figures 6A, 6B) [8].

TOF MR venography is the most commonly used method for CVT diagnosis. According to the time interval between the start of thrombus formation and the moment of imaging, different signal intensities are obtained in the thrombus in T1- and T2-weighted MR images due to the physiological degradation of hemoglobin and its paramagnetic effects [8]. In the subacute stage of thrombus development (six to 15 days), the signal is predominantly hyperintense on both T1- and T2WI because of methemoglobin in the thrombus [9]. The signal in a chronic thrombus is often isointense on T1- and T2-weighted MRI images, but could also be hyperintense on T2-weighted images. However, it should be noted that variability exists in each case and is usually significant [10].

In the first hours or days, the change in the thrombus signal can be so subtle that it can be difficult to identify. In this case, the GRE sequences are essential for the diagnosis [11]. In up to 41% of patients undergoing MRI studies due to the presence of a venous thrombus, an increase in intensity is identified in the diffusion sequences; this finding must always be corroborated in the ADC value, which decreases [12].

Between approximately six days and 15 days after thrombus formation, there may be a marked increase in signal intensity within the venous sinus, and this may be misinterpreted as the presence of flow on TOF MR venogram sequences. A careful and close evaluation usually allows differentiation, as the thrombus signal is typically not as intense as the flow-related signal. Also, T1WI in such cases depicts an abnormal increase in signal intensity within the sinus [13].

Arterial thrombosis

Arterial cerebral thrombosis occurs when blood flow to the brain is suddenly cut off due to vessel occlusion. Clinical presentation often includes slurred speech, unilateral face dropping, and weakness on one side of the body. Arterial thrombosis usually affects people with underlying atherosclerosis. Fatty deposits cause the arteries to harden and narrow over time and increase the risk of blood clots. The mechanism of thrombus formation in atherosclerotic cerebral arteries is still controversial, although intraplaque hemorrhage and rupture of the atheromatous plaques have been proposed. MRI has high sensitivity and specificity for this diagnosis, being able to detect well-defined periods of time and thus regulate therapeutic behavior [14,15].

**Figure 7:**
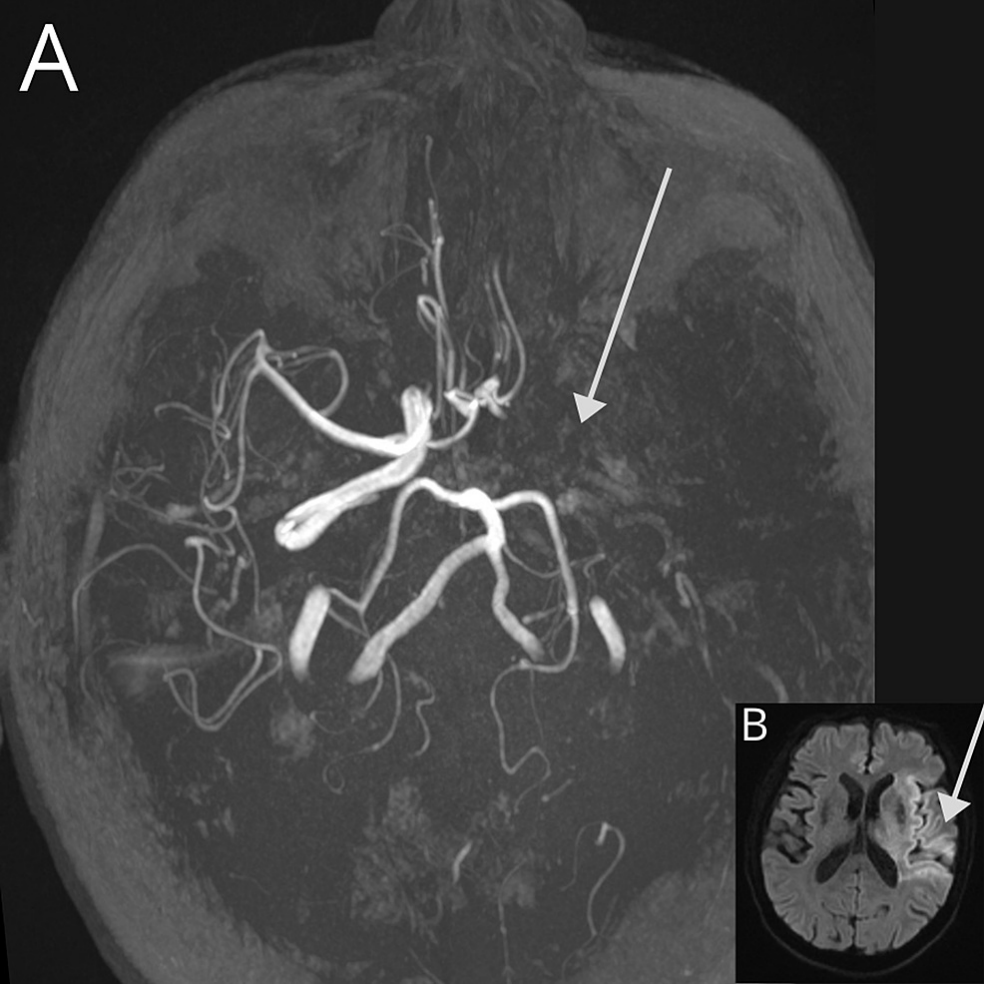
Arterial thrombosis in an 84-year-old male patient with a history of hypertension and outpatient management for COVID-19 (SARS-CoV-2 RT-PCR (+) test result) presented with sudden left hemiplegia, dysphagia, and motor aphasia. A CT scan was performed (normal) and MRI revealed the following: (A) 3D TOF on maximun intensity projection demonstrates the absence of flow signal on the left internal carotid artery, middle cerebral artery, and proximal anterior communicating artery; collateral circulation must supply the left anterior cerebral artery. (B) DWI with a hyperacute ischemic lesion (diffusion restriction) in the insular, frontal, and parietal lobe. COVID-19: coronavirus disease 2019; SARS-CoV-2 RT-PCR (+): positive severe acute respiratory syndrome coronavirus 2 reverse transcriptase-polymerase chain reaction; 3D TOF: three-dimensional time of light; DWI: diffusion-weighted imaging

In early hyperacute thrombosis (zero to six hours), DWI demonstrates reduced ADC values and an increased DWI signal. If infarction is incomplete, cortical contrast enhancement may be seen as early as two to four hours. In late hyperacute (six to 24 hours) thrombosis, a high T2WI signal will be detected on T2-FLAIR images (more easily seen) or conventional T2WI fast spin-echo [15,16]. 3D TOF revealed no flow (Figure 7A). An evolution of at least 16 hours from the formation of the thrombus allowed the observation of persistent hypointense images in the affected vessel in the T1 sequences. In acute thrombosis (24 hours to one week), the infarcted parenchyma continued to demonstrate a high DWI signal and low ADC signal (Figure 7B); however, by the end of the first week, ADC values start to increase. The infarct remained hyperintense on T2WI fast spin-echo (FSE) and T2-FLAIR, with the T2WI signal progressively increasing during the first four days. The T1WI signal remained low, although some cortical intrinsic high T1WI signals may be seen as early as three days after infarction. After day five, the cortex usually demonstrates contrast enhancement on T1WI. In the subacute stage of infarction (one to three weeks), ADC demonstrates "pseudo-normalization." The ADC values continue to increase as time passes, and the DWI signal for infarcted tissue remains elevated due to the persistent high T2 FSE/T2-FLAIR signal (T2 shine-through effect). This usually occurs between days 10 and 15. T1WI continued to show hypointensity with a cortical intrinsic high T1WI signal due to cortical laminar necrosis or pseudolaminar necrosis. In chronic infarction (more than three weeks), the T2WI signal was high, and the T1WI signal remained low with an intrinsic high T1WI signal in the cortex if cortical necrosis was present. From two to four months, the ADC values were higher than expected, and cortical contrast enhancement persisted [14,16].

Posterior reversible encephalopathy syndrome

Posterior reversible encephalopathy syndrome (PRES) refers to a clinical-radiological entity characterized by headaches, confusion, visual disturbances, seizures, and transient changes in brain substance detected with neuroimaging [17]. Patients most at risk are those with sepsis or shock associated with multiple organ dysfunction [18].

**Figure 8:**
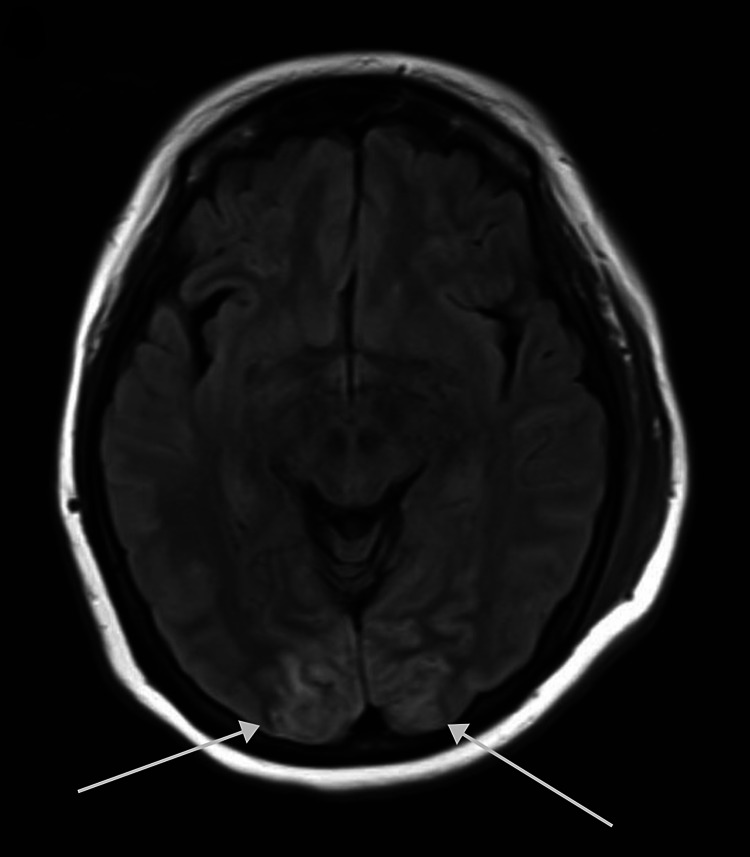
Posterior reversible encephalopathy syndrome in a 73-year-old woman with a history of rheumatoid arthritis and hypertension who was SARS-CoV-2 RT-PCR (+) with COVID-19 pneumonia and delirium. The figure shows cortico-subcortical occipital hyperintensities on the T2-FLAIR axial image. COVID-19: coronavirus disease 2019; SARS-CoV-2 RT-PCR (+): positive severe acute respiratory syndrome coronavirus 2 reverse transcriptase-polymerase chain reaction

The MRI pattern is often characteristic and represents an essential component of PRES diagnosis [17]. Typical lesions predominate in the posterior parietal or occipital lobe region white matter, with some involvement of the overlying cortex. This was the most consistent finding, seen in 98% of the patients, with additional involvement of the frontal lobes in 93%, which was typically linear along the superior frontal sulcus [18]. The lesions were hyperintense on T2‐weighted images and usually hypointense or isointense on diffusion‐weighted images, with an increase of the apparent diffusion coefficient indicating vasogenic edema (Figures 8, 4B, 4C) [17,18].

Metabolic cerebellar syndrome

This syndrome constitutes cerebellar abnormalities due to various toxic disorders, and various imaging findings can be seen, such as cerebellar hypoplasia, cerebellar atrophy, and patchy or diffuse white matter abnormalities, with the involvement of dentate nuclei and cerebellar cortex. Cerebellar atrophy can be differentiated from cerebellar hypoplasia by the presence of enlarged fissures in comparison to normal foliae secondary to loss of tissue in the former, but differentiation can be difficult at times, or atrophy can be superimposed on hypoplasia [19].

**Figure 9:**
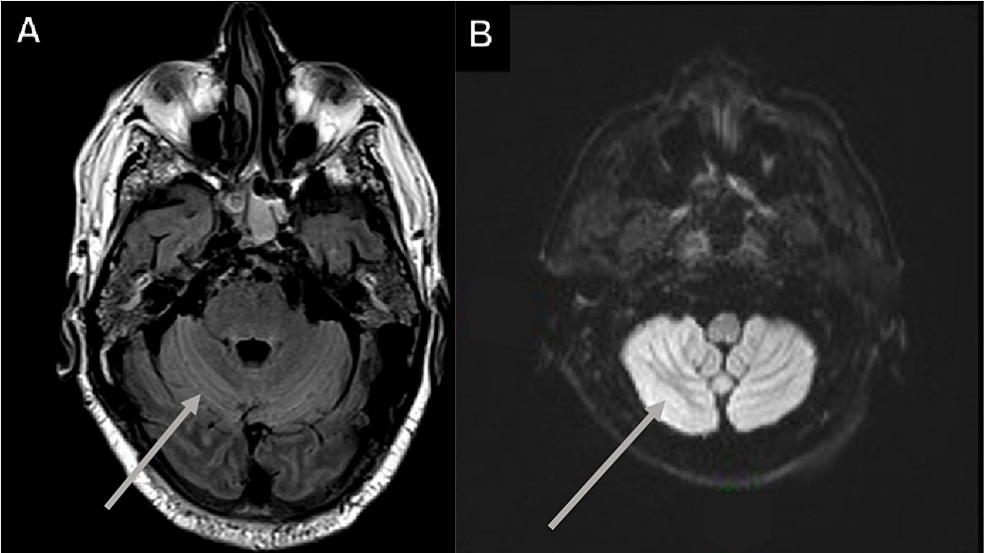
Metabolic cerebellar syndrome in a 47-year-old male with a history of dyslipidemia and symptomatic management for COVID-19 (SARS-CoV-2 RT-PCR {+}) development pneumonia and ataxia. (A and B) Increased cerebellar T2-FLAIR and DWI signal in a metabolic cerebellar syndrome. COVID-19: coronavirus disease 2019; SARS-CoV-2 RT-PCR (+): positive severe acute respiratory syndrome coronavirus 2 reverse transcriptase-polymerase chain reaction; T2-FLAIR: T2-weighted fluid-attenuated inversion recovery; DWI: diffusion-weighted imaging

MRI revealed an abnormal T2-hyperintense symmetric signal and nodularity within the cerebellar parenchyma. T2-FSE and T2-FLAIR hyperintensities that did not enhance were the most common finding. However, not all cases affected the dentate nuclei, with some cases demonstrating periventricular white matter involvement, cerebellar cortex or affecting the splenium and sparing the dentate nuclei (Figures 9A, 9B) [19,20].

Osmotic demyelination syndrome

Pontine myelinolysis is usually associated with electrolyte disturbances, which lead to encephalopathy or seizures. As normonatremia is restored and electrolyte values begin to normalize, the patient’s neurological and mental status improves and may return to normal within 48-72 hours, only to rapidly deteriorate days later. Symptoms during the second period of deterioration include dysarthria, dysphagia, flaccid quadriparesis that later becomes spastic, and progression to horizontal gaze paralysis, delirium, or coma [21]. Extrapontine myelinolysis can occur in cerebral white matter and basal ganglia, and less commonly in lateral geniculate bodies, hippocampi, and peripheral cortex [22]. It commonly occurs in conjunction with central pontine myelinolysis. To encompass both entities, the term osmotic demyelination syndrome is used [23].

**Figure 10:**
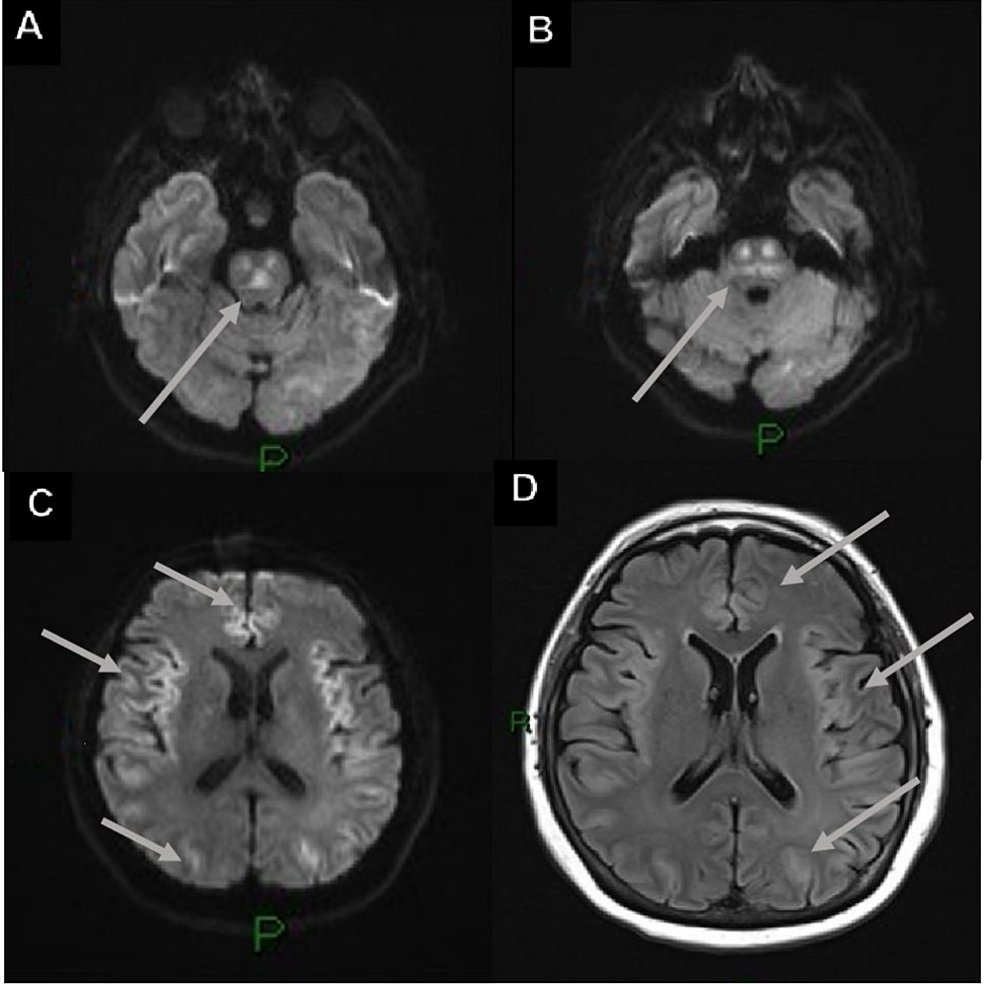
Osmotic demyelination syndrome in a 74-year-old male with a history of ischemic heart disease, abdominal compartmental syndrome, pulmonary thromboembolism, quadriplegia, and was also SARS-CoV-2 RT-PCR (+). (A and B) Axial DWI shows restricted diffusion in the pons with the trident sign. (C and D) DWI and T2-FLAIR show bilateral symmetrical restricted diffusion and vasogenic edema in the frontal, temporal, and occipital lobes. SARS-CoV-2 RT-PCR (+): positive severe acute respiratory syndrome coronavirus 2 reverse transcriptase-polymerase chain reaction; DWI: diffusion-weighted imaging; T2-FLAIR: T2-weighted fluid-attenuated inversion recovery

Affected regions demonstrated late low T1 signals and high T2 signals, but these changes can take up to two weeks to be observed using MRI. In the pons region, a classic trident shape was seen, and the piglet sign (likened to the face of a pig) was seen using T2-FLAIR axial MRI. Signal characteristics of the affected region included mild to moderate hypointensity on T1, hyperintensity on T2-FSE and T2-FLAIR, sparing of the peripheral and corticospinal tracts, and hyperintensity on DWI with low ADC (Figures 10A-10D) [24,25].

## Conclusions

The SARS-CoV-2 virus has tropism for different cells of the human body, including neurological ones (brainstem nuclei and glial cells), which explains the neurological symptoms of COVID-19. Neuroinfection occurs by hematogenous dissemination or a retrograde neuronal pathway and is characterized by a variety of signs and symptoms ranging from anosmia to loss of cardiorespiratory center function.

Key aspects of therapeutic development are the identification of the pathologies associated with this infection and those that are secondary to it, and the optimization of detection and diagnostic methods. Specific and global treatments can then be developed and administered to address the direct damage caused by the infection and reduce or eliminate the harmful effects of treatments.

MRI is the method of choice for detecting the described pathologies, but it has the disadvantage of acquisition times that can be complicated in an unstable patient. The long-term advantage is that it could open a line of research on determining long-term sequelae without the use of ionizing radiation and comprehensive rehabilitation. This could be justified by the current number of people presenting with a history of infection and progressive increases in reported sequelae (e.g., fatigue, anosmia, decreased attention span, and amnesia).
